# Behavioral scales variability in patients with prolonged disorders of consciousness

**DOI:** 10.1007/s10072-023-06812-x

**Published:** 2023-04-22

**Authors:** Maria Daniela Cortese, Martina Vatrano, Francesco Arcuri, Maria Girolama Raso, Paolo Tonin, Rocco Salvatore Calabrò, Francesco Riganello

**Affiliations:** 1 S. Anna Institute, Research in Advanced Neurorehabilitation, Via Siris 11, 88900 Crotone, Italy; 2 IRCCS Centro Neurolesi “Bonino Pulejo”, 98121 Messina, Italy

**Keywords:** WHIM, CRS-R, NCS, Prolonged disorders of consciousness, Unresponsive wakefulness syndrome, Minimally conscious state

## Abstract

**Background:**

The principal conditions differentiating disorders of consciousness (DOC) patients are the unresponsive wakefulness syndrome/vegetative state (UWS/VS) and the minimally conscious state (MCS). Many individuals who suffer from sudden-onset severe brain injury move through stages of UWS/VS and MCS before regaining full awareness. In some patients, the DOC condition is protracted for years (PDOC). In this study, we observed PDOC patients for 6 months to assess possible changes in their level of consciousness.

**Methods:**

We enrolled 40 PDOC patients, 23 UWS/VS and 17 MCS hosted in a dedicated unit for long-term brain injury care. The time from injury was 472 ± 533 days for UWS/VS and 1090 ± 1079 days for MCS. The Wessex Head Injury Matrix (WHIM), Coma Recovery Scale-R (CRS-R), and Nociception Coma Scale were administered monthly for 6 months.

**Results:**

During the period of assessment, the percentage of UWS/VS shifted from 58 to 45%, while for the MCS, from 42 to 55%. A positive correlation was found for the UWS/VS patients between the months of observation with the CRS-R total score and WHIM total numbers of behaviors (TNB). In the UWS/VS group, the CRS-R auditive and visual subscales correlated positively with the observation time. During the whole period of observation, 8 patients had constant CRS-R total scores while the WHIM TNB changed in 7 of them.

**Conclusion:**

Our findings demonstrated that the monthly assessment of PDOC by means of the CRS-R and WHIM was able to detect also subtle changes in consciousness level.

## Introduction

Consciousness is a complex concept that has several facets. It comes from the Latin *conscientia* that in turn derives from the verb *conscio*, *conscire*, and is created by the fusion of the preposition *cum* (with) and the verb *scio* (to know) [1]. The Latin root indicates the knowledge shared with another and, for extension, with oneself. The Oxford Companion to Philosophy states that “consciousness exists, but it resists definition” [2], implying the risk of being inaccurate when we define it.

With the term consciousness, we generally refer to the waking state (i.e., in the neurological field, consciousness is often associated with the waking state and with the ability to respond to stimuli in an integrated manner); to the perceptual awareness (i.e., to denote the perceptual awareness of a person or an animal); and to the intentional state (i.e., any mental state that has propositional content, such as a belief, fear, hope, expectation, or purpose) [3].

Among the several definitions of consciousness proposed [4], the one by William James [5] is helpful for describing disorders of consciousness (DOC) along a continuum: “at its least, normal human consciousness consists of a serially time-ordered, organized, restricted and reflective awareness of self and the environment. Moreover, it is an experience of graded complexity and quantity.” However, the intimate relationship between arousal level and the fundamental neuropsychological elements of normal conscious brain function, which supply the contents of consciousness, is omitted from this definition.

According to neurological studies, consciousness is characterized by two essential characteristics: wakefulness (i.e., the presence of spontaneous periods of opening the eyes) and awareness (i.e., the capacity of a subject to react to internal and external stimuli in an integrated manner) [6, 7]. A variety of disorders impacting one’s capacity to engage with the outside world are referred to as DOC [8], such as the unresponsive wakefulness syndrome/vegetative state (UWS/VS), the minimally conscious state (MCS), locked-in syndrome, and akinetic mutism [9, 10]. However, locked-in syndrome and akinetic mutism are different conditions from the DOC. The locked-in syndrome is characterized by quadriplegia, lower cranial nerve paralysis, and mutism. The consciousness is intact, but voluntary motor control is impaired except for some eye movements [11]. The akinetic mutism is characterized by diminished neurologic drive with a decrease in nearly all motor functions. Also, facial expressions, gestures, and speech output are impaired, but visual tracking is preserved [11]. The differential diagnosis among UWS/VS, MCS, akinetic mutism, and locked-in syndrome could not be as simple [10].

The principal conditions that differentiate UWS/VS and MCS are that the first is characterized by the spontaneous opening of the eyes and reflexive responses to external stimuli, while in the second [7, 12], the patients exhibit minimal but discernible signs of non-reflex behaviors, which occur reproducibly (yet inconsistently) as a response to visual, auditory, tactile, or noxious stimuli. Given the clinical heterogeneity of MCS patients, those that show an intelligible verbalization or gestural or verbal yes/no intentional communication, consistent command-following, and the presence of functional object use are generally classified as MCS + , while those without these criteria are classified MCS − [13, 14].

DOC can be caused by either traumatic (such as car accident) or non-traumatic (such as surgery, infection, anoxia, and cardiac arrest) events that cause widespread functional changes or by more widespread injuries.

Patients may or may not transition sequentially through each state of consciousness when experiencing DOC. Many individuals who suffer from sudden-onset severe brain injury move through UWS/VS and MCS before regaining full awareness. In some patients, this change may happen over a few days or weeks, while for others, the DOC condition is protracted for years (PDOC) [7, 15]. Some patients reach a plateau remaining the rest of their lives in UWS/VS or MCS [7]. It was observed that the restoration of cerebral network activity and consequent recovery of consciousness in PDOC patients [16], when present, could be slow [17, 18].

Accurate differential diagnosis is critical in the clinical management of DOC patients. The approach to treatment is driven by the diagnosis, which is strongly associated with functional outcome.

Diagnostic taxonomies based on pathophysiological mechanisms have yet to be developed, so DOC are classified primarily based on observable behavioral features and their inferred relationship to the level of consciousness.

Scales such as the Coma Recovery Scale-Revised (CRS-R) [19] and Wessex Head Injury Matrix (WHIM) [20] were developed to assess patients with DOC but using different approaches.

The CRS-R was developed to differentiate and diagnose UWS/VS, MCS conditions, and the emersion from MCS using the Aspen criteria [21]. Its scoring is based on the existence or absence of specific behavioral reactions (i.e., auditory, visual, motor, and oro/verbal functions, communication, and arousal) to standardized sensory stimuli. Following these criteria, a CRS-R total score higher than 8 indicates a higher probability of diagnosing an MCS, and a score of 10 or higher can be interpreted as a marker of conscious aware [22, 23]. Furthermore, in the CRS-R, some items are indicative of MCS (e.g., *fixation* in the visual function scale, *reproducible movement to command* in the auditory function scale, or *localization to noxious stimulation* in the motor function scale) or emersion from MCS (e.g., *functional object use* in the motor function scale).

Similarly, behavioral responses are used to assess pain in DOC patients. With this aim, the Nociception Coma Scale (NCS) [24] and its revised version (NCS-R) [25] were developed and used in assessing DOC patients, observing that a score of 5 and 3 for NCS and NCS-R, respectively, could be predictive of change of the level of consciousness from UWS/VS to MCS [26].

The WHIM does not directly distinguish UWS/VS and MCS patients but monitors subtle behavioral changes. It was developed to identify sequences of recovery processes encompassing cognitive, social, behavioral, attentive, and communicative aspects and is composed of 62 items and two scores that define the most advanced behavior (MAB) and total number of different behaviors items (TNB) [27].

In the CRS-R, the examiner, in line with the hierarchical organization of the scale, starts assessing the highest item (i.e., the item indicating contents of consciousness). The behavioral response to the stimulus must be repeated at least three times to assign the score. Then, once an item is scored, the examiner moves to the next subscale. The WHIM scale assesses behavioral responses with increasing complexity, and the score is based on the presence of the observed behavior.

The different approaches between CRS-R and WHIM in assessing the patients make the WHIM potentially more sensible in detecting subtle changes in PDOC patients.

In this study, we aim to observe the possible modification of the behavioral response in PDOC patients, with a time from injury of a minimum of 6 months, hosted in a dedicated care unit, and if the WHIM can help to detect subtle changes. With this aim, the behavioral responses were assessed by the CRS-R, WHIM, and NCS.

Considering the characteristics of the used scales, we expect to find a correlation between the behavioral scales and a higher sensitivity of the WHIM in observing a subtle change in the behavioral responses. When present, we also assume to find a correlation between a change in the level of consciousness assessed with CRS-R and WHIM and the observation period. Moreover, a higher variation in the MAB and TNB scores of the WHIM compared to the total score of the CRS-R and NCS is also conceivable.

## Materials and methods

### Patient population and setting

We enrolled 40 PDOC patients, 23 UWS/VS (8 female, age 56 ± 12; 15 male, age 54 ± 10) and 17 MCS (5 female, age 58 ± 11; 12 male 50 ± 18) with an educational level ranged between secondary school and graduate, hosted in the S. Anna Institute in a dedicated unit for long-term brain injury care. The time from injury was 472 ± 533 days for UWS/VS and 1090 ± 1079 days for MCS (Table 1).

**Table 1:** Demographics information

Patient	Diagnosis	Sex	Educational level	Age	Etiology	Time from injury (days)
1	UWS/VS	Male	H	62	TBI	490
2▲			G	47		394
3▲			S	62		258
4			H	56	HEM	297
5▲			G	51		295
6*^+^			S	65		273
7			G	59		968
8			G	52	ANOX	180
9			H	42		2291
10			G	61		197
11			S	70		187
12*^●^			H	39		257
13			H	64		214
14▲			H	47	OTHER	189
15▲			G	39		184
16	UWS/VS	Female	S	64	HEM	180
17			H	56		219
18			S	75		281
19*^+^			H	58		874
20			G	49		538
21*^●^			G	39	ANOX	190
22▲			H	65		1705
23			G	42		203
24	MCS	Male	H	27	TBI	411
25*			H	19		181
26			H	44		3012
27			S	69		2568
28			S	70		3325
29			G	47		1488
30			G	40		317
31			S	76	HEM	2143
32*			G	64		1021
33▼			H	41		277
34			H	66		193
35*^●^			G	42	ANOX	226
36	MCS	Female	S	65	HEM	196
37			H	48		366
38			G	47		1040
39			S	71		1591
40▼			H	58	OTHER	190

*UWS/VS*, unresponsive wakefulness syndrome; *MCS*, minimally conscious state; *TBI*, traumatic brain injury; *HEM*, hemorrhagic; *ANOX*, anoxic; *OTHER*, other etiology; ▲, change the level of consciousness in MCS; ▼, change the level of consciousness in UWS/VS; *discarged after 4 months; * + died after 4 months; *● discarged after 4 months and died in the successive months; *G*, graduate; *H*, high school; *S*, secondary school

Inclusion criteria were a diagnosis of UWS/VS or MCS based on the CRS-R and more than 180 days from the injury. Patients were excluded if they had clinical instability, sepsis, COVID-19 infection, and previous neurological or psychiatric disorders.

### Outcome measures and procedures

The patients were administered CRS-R, WHIM, and NCS once a month for 6 months (Tables 2, 3, and 4), for an overall time from the first assessment to the last of 5 months, by an expert rater with more than 15 years of experience treating DOC patients. The patients were assessed between 09:30 a.m. and 11:30 a.m., considering the fluctuation in the arousal [7] and following the indication in Candelieri [28] and Cortese [29], who observed a higher probability of obtaining a behavioral response in this time range. The assessment modality was the same across the patients and the time points. The time of administration of the WHIM takes around 30 min and never more than 40 min. Differently, the time of administration of the CRS-R lasted no more than 20 min and the NCS around 5 min.

**Table 2:** CRS-R assessment

		Total score (auditory/visual/motor/oromotor-verbal/communication/arousal)					
Patient	Diagnosis	Month					
		1	2	3	4	5	6
1	UWS/VS	7 (2/1/1/1/0/2)	7 (2/1/1/1/0/2)	7 (2/1/1/1/0/2)	7 (2/1/1/1/0/2)	7 (2/1/1/1/0/2)	7 (2/1/1/1/0/2)
2		6 (0/3/1/1/0/1)	12 (3/3/1/2/1/2)	9 (2/3/1/1/0/2)	10 (2/3/1/2/0/2)	9 (2/3/1/1/0/2)	10 (2/3/1/2/0/2)
3		5 (1/0/2/1/0/1)	10 (4/0/3/1/0/2)	9 (3/0/3/1/0/2)	10 (4/0/3/1/0/2)	10 (4/0/3/1/0/2)	9 (2/1/3/1/0/2)
4		7 (2/1/2/1/0/1)	7 (2/1/2/1/0/1)	7 (2/1/2/1/0/1)	7 (2/1/2/1/0/1)	5 (2/0/2/1/0/0)	6 (2/0/2/1/0/1)
5		6 (1/1/2/1/0/1)	6 (1/1/2/1/0/1)	9 (2/2/2/1/0/2)	9 (2/3/1/1/0/2)	10 (2/3/2/1/0/2)	10 (2/3/2/1/0/2)
6		6 (1/0/2/1/0/2)	6 (1/0/2/1/0/2)	6 (1/0/2/1/0/2)	6 (1/0/2/1/0/2)		
7		6 (1/0/2/1/0/2)	5 (1/0/2/1/0/1)	6 (1/0/2/1/0/2)	5 (1/0/2/1/0/1)	5 (1/0/2/1/0/1)	5 (1/0/2/1/0/1)
8		5 (1/0/2/1/0/1)	5 (1/0/2/1/0/1)	4 (1/0/2/1/0/0)	5 (1/0/2/1/0/1)	6 (1/0/2/1/0/2)	5 (1/0/2/1/0/1)
9		5 (1/1/1/0/0/2)	5 (1/1/1/0/0/2)	5 (1/1/1/0/0/2)	5 (1/1/1/0/0/2)	4 (1/1/1/0/0/1)	5 (1/1/1/0/0/2)
10		4 (1/0/1/1/0/1)	4 (1/0/1/1/1/0)	4 (1/0/1/1/0/1)	5 (1/0/1/1/0/2)	5 (1/0/2/1/0/1)	5 (1/0/2/1/0/1)
11		7 (2/0/2/1/0/2)	6 (1/0/2/1/0/2)	7 (2/0/2/1/0/2)	7 (2/0/2/1/0/2)	5 (1/0/2/1/0/1)	6 (1/0/2/1/0/2)
12		5 (1/0/2/1/0/1)	5 (1/0/2/1/0/1)	7 (1/1/2/1/0/2)	7 (1/1/2/1/0/2)		
13		4 (1/0/1/1/0/1)	5 (1/1/1/1/0/1)	6 (1/1/1/1/0/2)	6 (1/1/1/1/0/2)	5 (1/1/1/1/0/1)	5 (1/1/1/1/0/1)
14		8 (1/1/2/2/0/2)	8 (1/1/2/2/0/2)	11 (3/3/1/2/0/2)	11 (3/3/1/2/0/2)	9 (3/3/1/1/0/1)	9 (3/3/1/1/0/1)
15		4 (1/0/1/1/0/1)	10 (3/3/1/1/0/2)	11 (3/3/2/1/0/2)	12 (3/3/2/1/1/2)	16 (4/5/2/1/2/2)	17 (4/5/2/1/2/3)
16		4 (1/0/2/0/0/1)	5 (1/0/2/1/0/1)	5 (1/0/2/1/0/1)	5 (1/0/2/1/0/1)	5 (1/0/2/1/0/1)	6 (1/0/2/1/0/2)
17		6 (1/1/2/1/0/1)	6 (1/1/2/1/0/1)	6 (1/1/2/1/0/1)	8 (2/1/2/1/0/2)	8 (2/1/2/1/0/2)	8 (2/1/2/1/0/2)
18		6 (0/1/2/1/0/2)	6 (0/1/2/1/0/2)	6 (1/0/2/1/0/2)	6 (1/0/2/1/0/2)	6 (1/1/2/1/0/1)	6 (1/0/2/1/0/2)
19		6 (1/0/2/1/0/2)	6 (1/0/2/1/0/2)	6 (1/0/2/1/0/2)	6 (1/0/2/1/0/2)		
20		4 (1/0/1/1/0/1)	5 (1/0/1/1/0/2)	4 (1/0/1/1/0/1)	5 (1/0/1/1/0/2)	5 (1/0/1/1/0/2)	5 (1/0/1/1/0/2)
21		8 (2/1/2/1/0/2)	6 (1/0/2/1/0/2)	6 (1/0/2/1/0/2)	6 (1/0/2/1/0/2)		
22		7 (1/1/2/1/0/2)	9 (2/1/2/2/0/2)	9 (2/1/2/2/0/2)	9 (2/1/2/2/0/2)	9 (2/1/2/2/0/2)	9 (2/1/2/2/0/2)
23		6 (1/1/2/1/0/1)	6 (1/1/2/1/0/1)	8 (2/1/2/1/0/2)	8 (2/1/2/1/0/2)	8 (2/1/2/1/0/2)	8 (2/1/2/1/0/2)
24	MCS	10 (2/3/2/1/0/2)	10 (2/3/2/1/0/2)	10 (2/3/2/1/0/2)	11 (3/3/2/1/0/2)	11 (3/3/2/1/0/2)	11 (3/3/2/1/0/2)
25		9 (3/1/2/1/0/2)	11 (3/3/2/1/0/2)	11 (3/3/2/1/0/2)	11 (3/3/2/1/0/2)		
26		12 (0/3/5/2/0/2)	12 (0/3/5/2/0/2)	12 (0/3/5/2/0/2)	12 (0/3/5/2/0/2)	12 (0/3/5/2/0/2)	13 (0/4/5/2/0/2)
27		10 (2/3/2/1/0/2)	10 (2/3/2/1/0/2)	10 (2/3/2/1/0/2)	10 (2/3/2/1/0/2)	10 (2/3/2/1/0/2)	8 (1/3/2/1/0/1)
28		12 (2/2/5/1/0/2)	12 (2/2/5/1/0/2)	12 (2/2/5/1/0/2)	12 (2/2/5/1/0/2)	10 (2/1/5/1/0/1)	10 (2/0/5/1/0/2)
29		9 (2/3/1/1/0/2)	10 (2/3/2/1/0/2)	10 (2/3/2/1/0/2)	10 (2/3/2/1/0/2)	10 (2/3/2/1/0/2)	10 (2/3/2/1/0/2)
30		9 (2/2/2/1/0/2)	9 (2/2/2/1/0/2)	10 (2/3/2/1/0/2)	9 (2/2/2/1/0/2)	10 (2/3/2/1/0/2)	10 (2/3/2/1/0/2)
31		10 (2/3/2/1/0/2)	10 (2/3/2/1/0/2)	10 (2/3/2/1/0/2)	10 (2/3/2/1/0/2)	10 (2/3/2/1/0/2)	10 (2/3/2/1/0/2)
32		13 (2/3/4/2/0/2)	13 (2/3/4/2/0/2)	13 (2/3/4/2/0/2)	13 (2/3/4/2/0/2)		
33		10 (3/3/1/1/0/2)	4 (1/0/1/1/0/1)	4 (1/0/1/1/0/1)	3 (1/0/1/1/0/0)	5 (1/0/1/1/0/2)	7 (2/1/1/1/0/2)
34*		12 (3/3/3/1/0/2)	17 (4/5/5/1/0/2)	17 (4/5/5/1/0/2)	16 (4/3/5/2/0/2)	16 (4/3/5/2/0/2)	15 (3/3/5/2/0/2)
35		10 (2/3/1/2/0/2)	10 (2/3/1/2/0/2)	10 (2/3/1/2/0/2)	10 (2/3/1/2/0/2)		
36		9 (2/3/2/1/0/1)	9 (2/3/2/1/0/1)	9 (2/3/2/1/0/1)	10 (2/3/2/1/0/2)	10 (2/3/2/1/0/2)	10 (2/3/2/1/0/2)
37**		15 (4/3/5/2/0/1)	10 (2/3/2/1/0/2)	12 (3/3/2/2/0/2)	15 (3/4/5/1/0/2)	15 (3/4/4/2/0/2)	16 (3/5/4/2/0/2)
38		10 (2/3/2/1/0/2)	10 (2/3/2/1/0/2)	10 (2/3/2/1/0/2)	10 (2/3/2/1/0/2)	10 (2/3/2/1/0/2)	10 (2/3/2/1/0/2)
39		11 (2/3/3/1/0/2)	12 (2/3/3/2/0/2)	11 (2/3/3/2/0/1)	13 (2/3/4/2/0/2)	13 (2/3/4/2/0/2)	13 (2/3/4/2/0/2)
40		9 (3/3/1/1/0/1)	6 (1/0/1/2/0/2)	7 (2/1/1/1/0/2)	6 (1/1/1/1/0/2)	5 (1/0/1/1/0/2)	5 (1/0/1/1/0/2)

*UWS/VS*, unresponsive wakefulness syndrome/vegetative state; *MCS*, minimally conscious state

^*^MCS *plus*

^**^MCS *plus* with a fluctuation to *minus* at the second assessment and returning to *plus* at the other assessment

MCS minus are characterized by *visual fixation* and *pursuit*, *automatic motor reactions* (e.g., scratching, pulling the bed sheet) and *localization to noxious stimulation*. MCS plus has in addition: *follow simple commands*, *intelligibly verbalize* or *intentionally communicate* [14]

**Table 3:** NCS assessment

		Total score (motor/verbal/visual/facial)					
Patient	Diagnosis	Months					
		1	2	3	4	5	6
1	UWS/VS	4 (1/1/1/1)	5 (1/1/1/2)	4 (2/1/0/1)	4 (1/0/1/2)	4 (1/0/1/2)	4 (1/0/1/2)
2		2 (1/0/1/0)	3 (1/0/1/1)	3 (1/0/1/1)	3 (1/0/1/1)	3 (1/0/1/1)	3 (1/0/1/1)
3		3 (2/0/0/1)	6 (3/0/2/1)	6 (3/0/2/1)	6 (3/0/2/1)	6 (3/0/2/1)	6 (3/0/2/1)
4		3 (2/0/0/1)	3 (1/0/0/2)	3 (1/0/0/2)	4 (2/0/0/2)	4 (2/0/0/2)	4 (2/0/0/2)
5		1 (1/0/0/0)	2 (2/0/0/0)	3 (2/0/1/0)	5 (1/0/2/2)	5 (2/0/2/1)	5 (2/0/2/1)
6		8 (2/2/2/2)	8 (2/2/2/2)	8 (2/2/2/2)	8 (2/2/2/2)		
7		2 (1/0/0/1)	2 (1/0/0/1)	2 (1/0/0/1)	2 (1/0/0/1)	2 (1/0/0/1)	2 (1/0/0/1)
8		4 (2/0/1/1)	2 (2/0/0/0)	2 (2/0/0/0)	2 (2/0/0/0)	3 (2/0/1/0)	3 (2/0/1/0)
9		2 (1/0/0/1)	2 (1/0/0/1)	2 (1/0/0/1)	2 (1/0/0/1)	2 (1/0/0/1)	2 (1/0/0/1)
10		2 (1/0/1/0)	3 (1/0/1/1)	2 (1/0/1/0)	3 (1/0/1/1)	3 (1/0/1/1)	3 (1/0/1/1)
11		5 (2/0/1/2)	5 (2/0/1/2)	5 (2/0/1/2)	5 (2/0/1/2)	5 (2/0/1/2)	4 (2/0/1/1)
12		3 (2/0/0/1)	4 (2/0/0/2)	2 (2/0/0/0)	2 (2/0/0/0)		
13		2 (1/0/0/1)	3 (1/0/1/1)	3 (1/0/1/1)	3 (1/0/1/1)	3 (1/0/1/1)	3 (1/0/1/1)
14		7 (2/2/1/2)	7 (2/2/1/2)	6 (1/2/1/2)	6 (1/2/1/2)	4 (1/1/1/1)	4 (1/1/1/1)
15		1 (1/0/0/0)	2 (2/0/0/0)	3 (2/0/1/0)	5 (2/0/2/1)	7 (2/0/3/2)	7 (2/0/3/2)
16		3 (2/0/1/0)	3 (2/0/1/0)	3 (2/0/1/0)	3 (2/0/0/1)	3 (2/0/0/1)	3 (2/0/0/1)
17		5 (2/0/1/2)	5 (2/0/1/2)	5 (2/0/1/2)	5 (2/0/1/2)	5 (2/0/1/2)	4 (2/0/0/2)
18		4 (2/0/1/1)	5 (2/0/2/1)	5 (2/0/2/1)	5 (2/0/2/1)	5 (2/0/2/1)	5 (2/0/2/1)
19		4 (2/0/1/1)	4 (2/0/1/1)	4 (2/0/1/1)	4 (2/0/1/1)		
20		4 (2/0/1/1)	4 (2/0/1/1)	4 (2/0/1/1)	4 (2/0/1/1)	4 (2/0/1/1)	4 (2/0/1/1)
21		3 (2/0/0/1)	4 (2/0/0/2)	5 (2/0/1/2)	5 (2/0/1/2)		
22		6 (2/1/1/2)	6 (2/1/1/2)	5 (2/0/1/2)	5 (2/0/1/2)	6 (2/1/1/2)	5 (2/0/1/2)
23		5 (2/0/1/2)	5 (2/0/1/2)	5 (2/0/1/2)	5 (2/0/1/2)	5 (2/0/1/2)	5 (2/0/1/2)
24	MCS	6 (2/0/2/2)	6 (2/0/2/2)	5 (2/0/1/2)	5 (2/0/1/2)	6 (2/0/2/2)	4 (2/0/2/0)
25		3 (2/0/0/1)	3 (2/0/0/1)	3 (2/0/0/1)	3 (2/0/0/1)		
26		7 (2/1/2/2)	7 (2/1/2/2)	7 (2/1/2/2)	7 (2/1/2/2)	7 (2/1/2/2)	4 (2/1/1/0)
27		4 (2/0/1/1)	5 (2/0/2/1)	5 (2/1/1/1)	5 (2/0/2/1)	5 (2/0/2/1)	5 (2/0/2/1)
28		5 (2/0/1/2)	5 (2/0/1/2)	5 (2/0/1/2)	5 (2/0/1/2)	4 (2/0/1/1)	7 (2/2/1/2)
29		3 (2/0/0/1)	3 (2/0/0/1)	3 (2/0/0/1)	3 (2/0/0/1)	3 (2/0/0/1)	3 (2/0/0/1)
30		6 (2/1/1/2)	7 (2/1/1/3)	7 (2/1/1/3)	6 (2/1/1/2)	7 (2/1/1/3)	7 (2/1/1/3)
31		4 (1/0/1/2)	4 (1/0/1/2)	4 (1/0/1/2)	4 (1/0/1/2)	4 (1/0/1/2)	4 (1/0/1/2)
32		6 (2/0/2/2)	7 (2/0/3/2)	6 (2/0/2/2)	7 (2/0/3/2)		
33		5 (1/0/2/2)	2 (1/0/1/0)	2 (1/0/1/0)	1 (1/0/0/0)	1 (1/0/0/0)	2 (1/0/1/0)
34		5 (3/0/1/1)	5 (2/0/2/1)	5 (2/0/2/1)	7 (2/0/3/2)	7 (2/0/3/2)	7 (2/0/3/2)
35		7 (1/2/2/2)	8 (2/2/2/2)	8 (2/2/2/2)	8 (2/2/2/2)		
36		7 (2/0/2/3)	7 (2/0/2/3)	7 (2/0/2/3)	7 (2/0/2/3)	6 (2/0/2/2)	4 (2/0/1/1)
37		6 (2/0/3/1)	6 (2/0/3/1)	5 (2/0/2/1)	6 (2/0/3/1)	8 (2/1/3/2)	8 (2/1/3/2)
38		5 (2/0/1/2)	5 (2/0/1/2)	5 (2/0/1/2)	5 (2/0/1/2)	6 (2/0/2/2)	6 (2/0/2/2)
39		10 (3/2/3/2)	8 (3/1/2/2)	8 (3/1/2/2)	8 (3/1/2/2)	8 (3/1/2/2)	8 (3/1/2/2)
40		3 (1/0/1/1)	3 (1/0/1/1)	2 (1/0/1/0)	2 (1/0/1/0)	2 (1/0/0/1)	3 (1/0/1/1)

*UWS/VS*, unresponsive wakefulness syndrome/vegetative state; *MCS*, minimally conscious state

**Table 4:** WHIM assessment

MAB/TNB TNB-clusters: (vigilance-attention/auditive/visulal/oroverbal/communication)							
patient	Diagnosis	Months					
		1	2	3	4	5	6
1	UWS/VS	20/7 (2/0/2/3/0)	20/7 (2/0/2/3/0)	20/7 (2/0/2/3/0)	7/4 (2/0/1/1/0)	7/4 (2/0/1/1/0)	7/4 (2/0/1/1/0)
2		26/8 (2/0/3/2/1)	34/19 (2/2/8/4/3)	30/17 (2/2/9/4/0)	30/17 (2/2/9/4/0)	30/17 (2/2/9/4/0)	30/17 (2/2/9/4/0)
3		7/3 (2/0/0/1/0)	22/9 (2/2/3/1/1)	22/9 (2/2/3/1/1)	22/9 (2/2/3/1/1)	22/9 (2/2/3/1/1)	16/7 (2/0/3/2/0)
4		14/6 (3/0/0/3/0)	7/4 (2/0/1/1/0)	7/4 (2/0/1/1/0)	23/9 (2/2/3/1/1)	23/9 (2/2/3/1/1)	23/9 (2/2/3/1/1)
5		22/9 (2/1/5/1/0)	22/6 (2/0/3/1/0)	28/10 (2/3/5/1/0)	22/11 (2/1/7/1/0)	22/12 (3/1/7/1/0)	22/12 (2/2/7/1/0)
6		13/5 (2/0/2/1/0)	7/4 (2/0/1/1/0)	7/4 (2/0/1/1/0)	7/4 (2/0/1/1/0)		
7		12/5 (2/0/2/1/0)	12/6 (2/0/2/2/0)	14/5 (2/0/2/1/0)	14/6 (2/0/2/2/0)	14/6 (2/0/2/2/0)	14/6 (2/0/2/2/0)
8		4/2 (2/0/0/0/0)	4/2 (2/0/0/0/0)	4/2 (2/0/0/0/0)	7/3 (2/0/0/1/0)	13/5 (2/0/2/1/0)	7/4 (2/0/1/1/0)
9		7/3 (1/0/1/1/0)	7/3 (1/0/1/1/0)	7/3 (1/0/1/1/0)	7/3 (1/0/1/1/0)	7/3 (1/0/1/1/0)	7/3 (1/0/1/1/0)
10		7/3 (1/0/1/1/0)	7/4 (2/0/1/1/0)	7/4 (2/0/1/1/0)	7/4 (2/0/1/1/0)	7/4 (2/0/1/1/0)	7/4 (2/0/1/1/0)
11		14/7 (3/0/2/2/0)	14/7 (3/0/2/2/0)	14/7 (3/0/2/2/0)	14/7 (3/0/2/2/0)	14/7 (3/0/2/2/0)	23/6 (2/0/1/2/1)
12		15/6 (2/1/2/1/0)	15/5 (2/1/1/1/0)	15/5 (2/1/1/1/0)	15/5 (2/1/1/1/0)		
13		12/4 (2/0/1/1/0)	12/5 (2/0/2/1/0)	12/5 (2/0/2/1/0)	12/5 (2/0/2/1/0)	12/5 (2/0/2/1/0)	12/5 (2/0/2/1/0)
14		8/6 (2/0/2/2/0)	23/16 (2/2/7/4/1)	36/17 (2/2/8/3/2)	36/18 (2/2/8/4/2)	24/11 (2/1/6/2/0)	24/11 (2/1/6/2/0)
15		7/3 (2/0/0/1/0)	28/13 (2/3/7/2/0)	28/15 (2/3/9/2/0)	41/22 (4/5/13/1/1)	41/23 (4/5/13/2/1)	41/22 (4/5/13/1/1)
16		7/4 (2/0/1/1/0)	13/4 (2/0/1/1/0)	13/4 (2/0/1/1/0)	7/4 (2/0/1/1/0)	7/4 (2/0/1/1/0)	7/4 (2/0/1/1/0)
17		11/4 (2/0/1/1/0)	11/5 (3/0/1/1/0)	11/5 (3/0/1/1/0)	11/5 (3/0/1/1/0)	11/5 (3/0/1/1/0)	11/5 (3/0/1/1/0)
18		7/4 (2/0/1/1/0)	7/4 (2/0/1/1/0)	8/5 (2/0/2/1/0)	14/5 (2/0/1/2/0)	14/5 (2/0/1/2/0)	7/4 (2/0/1/1/0)
19		14/4 (2/0/1/1/0)	4/3 (2/0/1/0/0)	4/3 (2/0/1/0/0)	4/3 (2/0/1/0/0)		
20		14/5 (2/0/1/2/0)	14/5 (2/0/1/2/0)	7/4 (2/0/1/1/0)	7/4 (2/0/1/1/0)	7/4 (2/0/1/1/0)	7/4 (2/0/1/1/0)
21		13/3 (2/0/0/1/0)	13/4 (2/0/1/1/0)	15/6 (2/1/2/1/0)	15/6 (2/1/2/1/0)		
22		20/7 (2/0/2/3/0)	22/8 (2/1/2/3/0)	22/13 (2/1/6/4/0)	22/14 (2/1/6/4/1)	22/12 (2/1/4/4/1)	22/10 (2/1/3/4/0)
23		23/6 (2/0/2/1/1)	23/6 (2/0/2/1/1)	23/6 (2/0/2/1/1)	23/6 (2/0/2/1/1)	23/6 (2/0/2/1/1)	23/6 (2/0/2/1/1)
24	MCS	24/12 (2/1/7/1/1)	24/12 (2/1/7/1/1)	22/12 (2/2/7/1/0)	22/12 (2/2/7/1/0)	22/13 (2/2/8/1/0)	22/11 (2/2/6/1/0)
25		15/5 (2/1/1/1/0)	18/8 (2/1/4/1/0)	25/16 (3/2/7/2/2)	25/16 (3/2/7/2/2)		
26		38/19 (3/0/11/4/1)	38/19 (3/0/11/4/1)	38/19 (3/0/11/4/1)	36/18 (3/0/10/4/1)	36/18 (3/0/10/4/1)	35/13 (2/0/8/3/0)
27		22/10 (2/1/6/1/0)	22/11 (2/1/7/1/0)	22/15 (2/2/8/3/0)	22/14 (2/2/8/2/0)	22/12 (2/2/7/1/0)	22/12 (2/2/7/1/0)
28		31/10 (2/1/4/2/1)	31/10 (2/1/4/2/1)	31/10 (2/1/4/2/1)	31/10 (2/1/4/2/1)	31/9 (2/1/4/1/1)	22/10 (2/1/3/4/0)
29		24/12 (3/0/6/2/1)	24/13 (3/1/6/2/1)	24/13 (3/1/6/2/1)	24/13 (3/1/6/2/1)	24/13 (3/1/6/2/1)	24/13 (3/1/6/2/1)
30		24/15 (1/2/6/5/1)	24/15 (1/2/6/5/1)	24/15 (1/2/6/5/1)	24/15 (1/2/6/5/1)	24/15 (1/2/6/5/1)	24/15 (1/2/6/5/1)
31		23/10 (3/0/5/1/1)	23/10 (3/0/5/1/1)	23/10 (3/0/5/1/1)	23/10 (3/0/5/1/1)	23/10 (3/0/5/1/1)	23/10 (3/0/5/1/1)
32		36/20 (2/3/10/4/1)	36/19 (2/3/10/3/1)	36/20 (2/3/10/4/1)	36/19 (2/3/10/3/1)		
33		24/13 (2/2/8/1/0)	7/3 (2/0/0/1/0)	7/3 (2/0/0/1/0)	7/4 (2/0/1/1/0)	7/5 (2/0/2/1/0)	13/8 (3/0/4/1/0)
34		29/12 (2/3/6/3/0)	33/16 (2/4/9/3/0)	33/16 (2/4/9/3/0)	33/18 (2/3/9/5/0)	36/22 (2/4/11/4/1)	36/21 (2/4/12/4/1)
35		24/14 (2/2/7/2/1)	24/14 (2/2/7/2/1)	36/18 (2/2/10/3/1)	36/18 (2/2/10/3/1)		
36		22/12 (2/1/6/2/1)	22/13 (2/1/6/3/1)	22/13 (2/1/6/3/1)	22/13 (2/1/6/3/1)	22/13 (2/1/6/3/1)	22/8 (2/1/4/1/0)
37		23/16 (2/2/7/4/1)	23/16 (2/2/7/4/1)	29/17 (2/4/9/3/1)	29/17 (2/4/9/3/1)	29/17 (2/4/9/3/1)	29/17 (2/4/9/3/1)
38		22/9 (1/1/5/2/0)	35/15 (2/1/10/1/1)	35/16 (2/1/10/1/2)	35/14 (2/1/9/1/1)	36/16 (2/1/10/1/2)	36/16 (2/1/10/1/2)
39		40/25 (4/3/12/4/2)	40/25 (4/1/11/3/6)	40/25 (4/1/11/3/6)	40/25 (4/1/11/3/6)	40/25 (4/1/11/3/6)	39/25 (4/1/11/4/5)
40		24/9 (3/0/4/2/0)	24/9 (3/0/4/2/0)	16/5 (2/0/2/1/0)	16/4 (2/0/1/1/0)	16/4 (2/0/1/1/0)	16/4 (2/0/1/1/0)

*UWS/VS*, unresponsive wakefulness syndrome/vegetative state; *MCS*, minimally conscious state; *MAB*, most advanced behaviors; *TNB*, total numbers of different behaviors; *TNB-cluster*, total numbers of different behaviors in the cluster subdivision

The CRS-R consists of 23 items divided into six subscales (i.e., auditory, visual, motor, oromotor/verbal, communication, and arousal subscales), which are arranged hierarchically. The scale assesses brain stem, cortical, and sub-cortical functioning. The scoring is based on the presence or absence of specific behavioral reactions to standardized sensory stimuli. The lowest items of the scale represent a reflexive activity, and the higher items a cognitively mediated response [19].

The WHIM is composed of 62 items hierarchically organized. The sequence is organized in a well-defined category of observation regarding the individual’s level of responsiveness and interaction with the environment. It was designed to observe spontaneous behaviors (e.g., opening eyes or attempting to remove a nasogastric tube), behavioral responses to environmental stimuli presented accidentally (e.g., turning the head briefly toward a noise), and to a standard set of stimuli (e.g., calling the patient’s name). All the observed behavior was marked with “ − ” if they met the operational definitions; otherwise were marked with “ + .” After ten consecutive not-observed behaviors, the assessment ended. The last marked item represents the MAB score, and the number of observed items is the TNB score [20].

NCS consists of 12 items divided into four subscales to assess motor, verbal, visual, and facial responses to noxious stimulation, while the revised version does not include the visual subscale. Each subscale ranges from 0 (no response) to 3 (appropriate response). The behavioral response was assessed by nailbed pressure applied to the four limbs for 5 s and ended as soon as a behavioral response was observed. Behavioral responses were recorded for 10 s after each noxious stimulus. The best-observed response was used to assign the score [24].

### Statistical analyses

The correlation between the CRS-R and NCS total score WHIM TNB and MAB and the correlation between months of assessment and scales were explored by the Spearman correlation test.

**Fig. 1:**
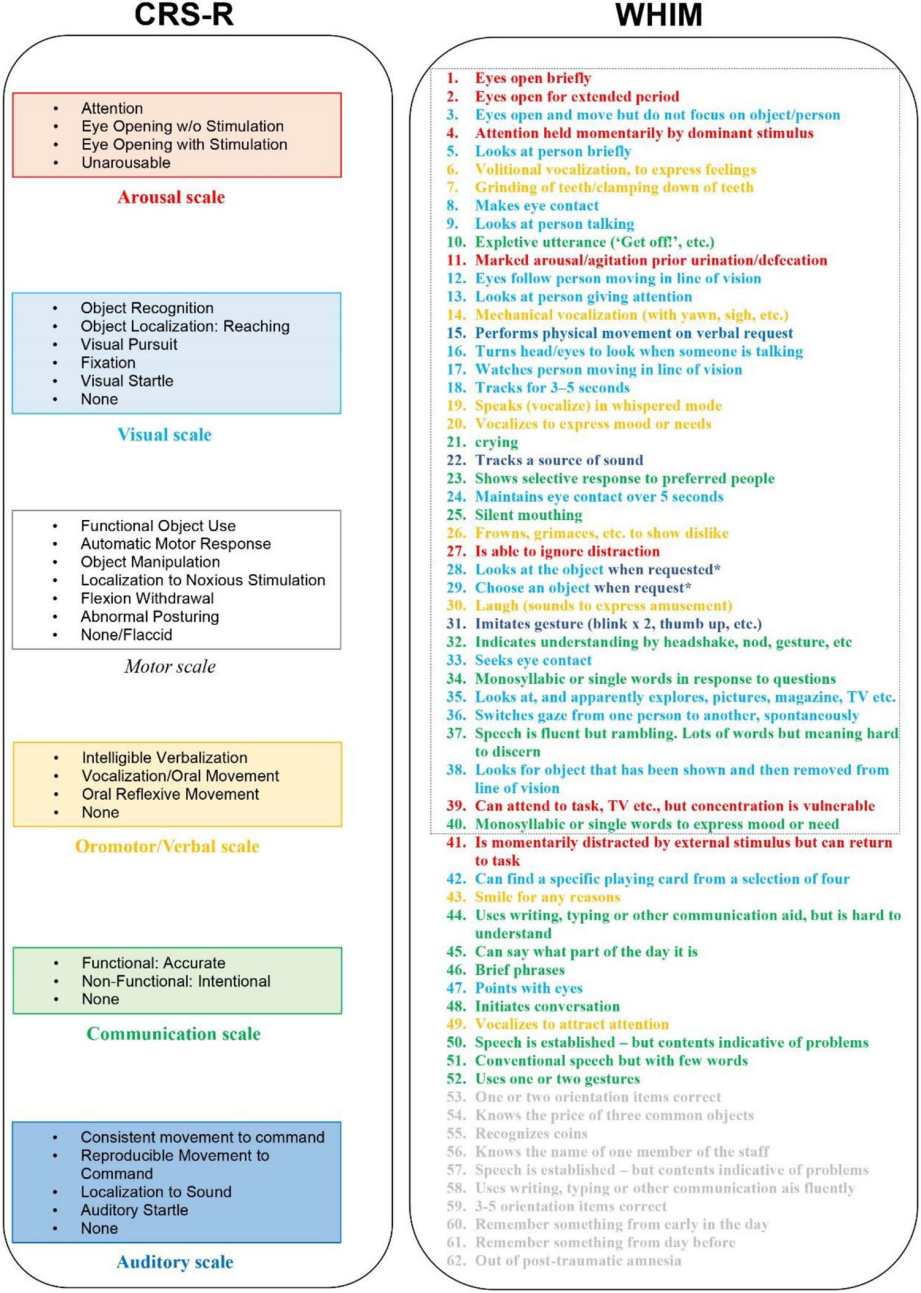
On the left are the subscales of the CRS-R; on the right are the WHIM items (see Table 5). The color represents the WHIM items’ clusterization based on the CRS-R subscales. In red, the vigilance-attention items (CRS-R arousal subscale); in light blue, the visual items (CRS-R visual subscale); in yellow, the oro-verbal items (CRS-R oromotor/verbal subscale); in green, the communication items (CRS-R communication subscale); in blue, the auditory items (CRS-R auditory subscale); in gray, the items that are not possible to cluster following the CRS-R subscales. Items 28 and 29 are in the visual and auditory clusters because they have characteristics of both the CRS-R subscales. In the dashed box, the items observed in the patient’s group

The correlation between the months of observation and the CRS-R, NCS, and WHIM subscales was also explored. We additionally clustered the WHIM’s items following the criteria of the CRS-R in TNB visual, auditive, arousal, oromotor/verbal, and communication subscales (Fig. 1, Table 5) to assess the correlation between each behavioral function and period of observation as assessed for the CRS-R.

**Table 5:** Cluster of WHIM items following the CRS-R criteria

			WHIM items
Cluster	CRS-R subscales	Auditive	15, 22, 28, 29, 31
		Visual	3, 5, 8, 9, 12, 13, 16, 17, 18, 24, 28, 29, 33, 35, 36, 38, 42, 47
		Oro-verbal	6, 7, 14, 19, 20, 26, 30, 43, 49
		Communication	10, 21, 23, 25, 32, 34, 37, 40, 44, 45, 46, 48, 50, 51, 52
		Arousal/attention	1, 2, 4, 11, 27, 39, 41
		Motor	n/a
		Other	53, 54, 55, 56, 57, 58, 59, 60, 61, 62

Variation between the level of consciousness between the first assessment and the successive ones was compared by paired *t*-test. For multiple comparisons, the *p*-value was set to 0.005.

The study was approved by the Ethical Committee (Regione Calabria Comitato Etico Sezione Area Centro, n.320, December 21, 2017). The patients’ relatives and caregivers were informed about the experimental procedure and gave their consent. The study was conducted according to the World Medical Association’s Declaration of Helsinki.

## Results

Comparing MCS and UWS/VS groups for age and gender, no differences were found. Significant differences were found for time from injury (*t*(38) = 2.17, *p* = 0.04) with higher values for MCS groups, and for the etiology (*Χ*^2^ = 8.5, *p* = 0.04) with higher number of traumatic brain injury (TBI) patients in MCS group and higher anoxic patients in UWS/VS group.

**Fig. 2:**
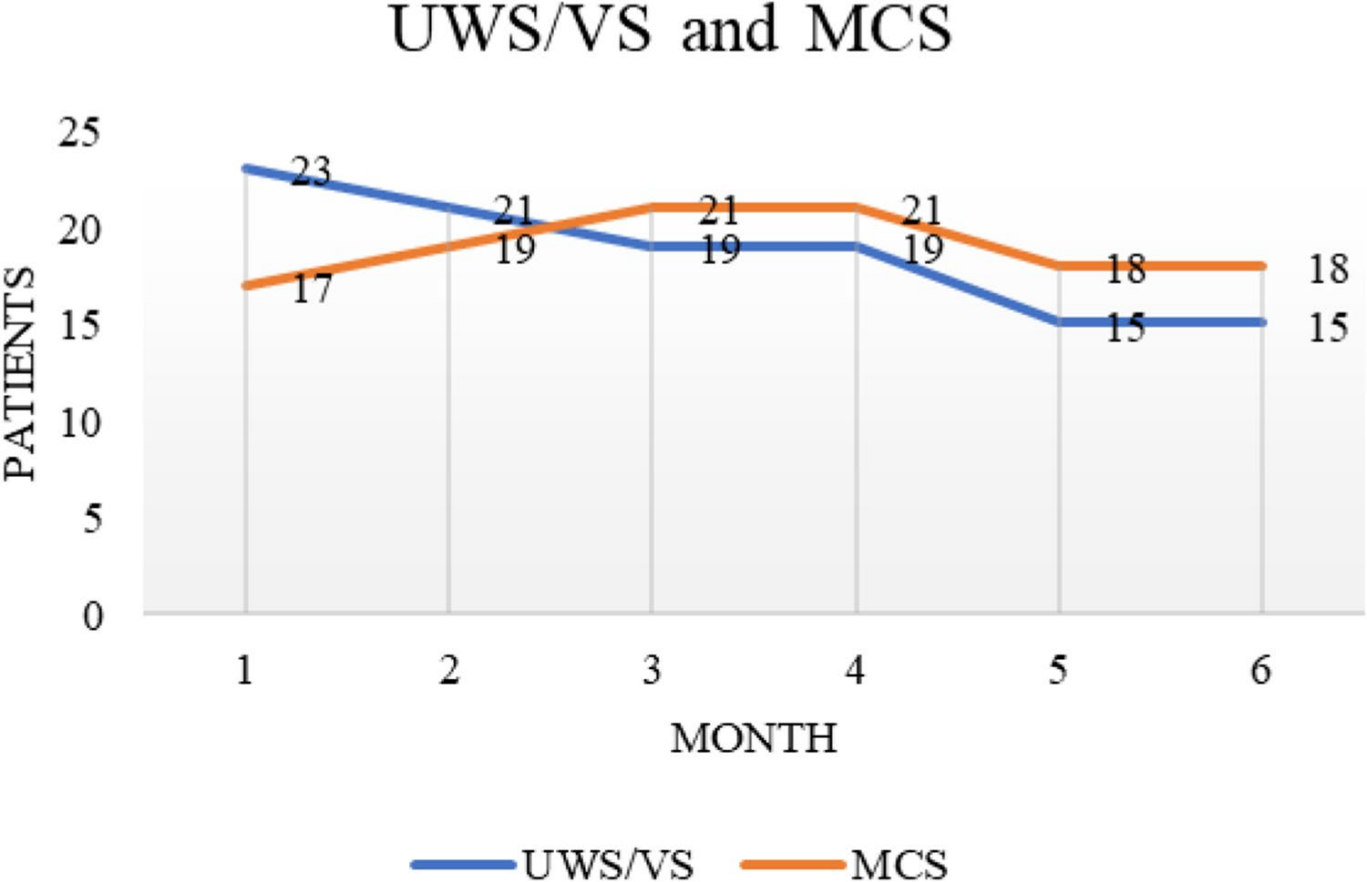
Number of UWS/VS and MCS patients during the months of observation. The number of patients is 40 from months 1 to 4 and 33 from 5 to 6

After 4 months, 3 MCS patients and 2 UWS/VS returned home, while 2 UWS/VS patients died. Six UWS/VS patients (i.e., 1 anoxic, 1 hemorrhagic, 2 traumatic brain injury, 2 other etiology) changed the level of consciousness in MCS, and 2 MCS patients (i.e., 1 hemorrhagic, 1 other etiology) in UWS/VS. For the patients who changed the diagnosis of the level of consciousness, the range from the acute event to the first assessment in the dedicated unit for long-term brain injury care was from 184 to 1705 days for the UWS/VS and from 190 to 277 days for the MCS. During the 6 months of assessment, the percentage of UWS/VS shifted from 58 to 45%, while for the MCS, from 42 to 55% (Fig. 2), without any significant differences between the first and the successive months of observation (Table 1).

At the Spearman correlation test, considering the diagnosis of consciousness level in the first month, all the total scores scales were correlated between them (all patients: 0.52 ≤ rho ≤ 0.92; *p* < 0.0001; MCS: 0.51 ≤ rho ≤ 0.81 *p* < 0.0001; UWS/VS 0.30 ≤ rho ≤ 0.92, 0.0005 ≤ *p* < 0.0001). A positive correlation was found for the UWS/VS patients between the months of observation with the CRS-R total score (rho = 0.19; *p* = 0.03) and WHIM TNB (rho = 0.18; *p* = 0.04), while no correlations were for the MCS patients. In UWS/VS group, the CRS-R auditive subscale correlated positively with the time of observation (rho = 0.28, *p* = 0.001). Furthermore, considering the WHIM, in the UWS/VS group, a positive correlation was found between time and cluster of auditive (rho = 0.18, *p* = 0.04) and visual (rho = 0.20; *p* = 0.02) items.

During the whole period of observation, 8 patients had constant CRS-R total scores (Table 2, n. 1, 6, 18, 19, 31, 32, 35, 38), while the WHIM TNB changed in 7 (Table 4, n. 1, 6, 18, 19, 32, 35, 38) and WHIM MAB changed in 6 of them (Table 4, n. 1, 6, 18, 19, 35, 38).

Considering the first four assessments, the CRS-R total score remained constant in 13 patients (Table 2, n. 1, 4, 6, 9, 18, 19, 26—28, 31, 32, 35, 38). In contrast, the WHIM TNB remains constant in 9 patients (Table 4, n. 9, 11, 16, 23, 24, 28, 30, 31, 39) and the WHIM MAB in 15 patients (Table 4, n. 9, 10–13, 17, 23, 27–32, 36, 39). Considering six assessments, the CRS-R total score was constant in 4 patients (Table 2, n. 1, 18, 31, 38). In comparison, the WHIM TNB was constant in 6 patients (Table 4, n. 9, 16, 23, 30, 31, 39) and WHIM MAB in 10 patients (Table 4, n. 9, 10, 13, 17, 23, 27, 29–31, 36).

Of the 13 patients with constant CRS-R total scores in the first 4 assessments, the WHIM TNB changed in 10 (Table 4, n. 1, 4, 6, 18, 19, 26, 27, 32, 35, 38) and WHIM MAB in 8 (Table 4, n. 1, 4, 6, 18, 19, 26, 35, 38) of them, while considering 6 assessments, the CRS-R total score was constant in 4 patients, and the WHIM TNB/MAB changed in 3 (Table 4, n. 1, 18, 38) of them.

For MCS and UWS/VS groups, comparing by the paired *t*-test the scales total scores and relatives subscales scores in the first month with the assessments in the successive months, no difference was found after Bonferroni’s correction.

## Discussion

Modern emergency treatments and life-support systems have significantly improved the treatment of severe head injuries due to traumatic or non-traumatic causes. However, 10–15% of these patients enter a condition known as DOC, which encompasses the syndromes of coma, UWS/VS, and MCS [7, 12]. PDOC refers to UWS/VS and MCS patients that remain in these pathological conditions for more than 4 weeks [7, 12]. These conditions may be transient, and some patients may progress from UWS to MCS before regaining full consciousness [7].

The patients that remain in UWS/VS after 4 weeks are classified as being in a persistent UWS/VS condition [30]. The diagnosis of the patients is permanent UWS/VS some months after a non-traumatic brain injury (i.e., three in the USA and six in the UK) or 1 year after a traumatic injury [30]. Others, however, have PDOC that will last the rest of their lives [12, 15].

In this study, we found that (1) some patients diagnosed with PDOC change their level of consciousness also after several years after the acute event; (2) also if the observed change in the level of consciousness regards few patients, it seems to be independent of the etiology of trauma and time from the injury; (3) CRS-R and WHIM well detect the change in the level of consciousness but the WHIM can detect subtle modification in the patients’ behavioral when the CRS-R remain constant. This latter point is the main finding and deserves further investigation and confirmation.

Indeed, it can be challenging to distinguish between reflexive and voluntary behaviors, and subtle signs of consciousness may go unnoticed, making it hard to distinguish MCS from UWS/VS. The established diagnostic criteria for MCS would reduce the incidence of misdiagnosis, but several recent studies found that about 40% of patients thought to be in UWS/VS are misdiagnosed [31]. The rate of misdiagnosis is similar also in PDOC patients [32]. Several factors can concur in formulating an incorrect diagnosis, such as the examiner’s experience. Fluctuation of arousal and lack of a series of assessments with sufficient observation time could hamper capturing the full range of behavior. Again, pain, motor impairment, cortical sensory deficits, and cognitive deficits, such as aphasia, could make it difficult to detect signs of consciousness [33].

It was observed how the presence of relatives and the use of familiar objects might reduce the misdiagnosis [34–37]. During the assessment, the inclusion of the relative can help detect changes in the clinical status, improving the patient’s diagnosis [10]. Sattin and colleagues highlighted the importance of caregivers’ presence in assessing patients with DOC and how they can contribute to the definition of the optimal setting for the behavioral evaluation of patients [38].

All these aspects should be considered in the assessment and rehabilitation of PDOC patients. Indeed, it was reported that, with appropriate treatment and specialized rehabilitation, two-thirds of patients with PDOC recovered consciousness after traumatic brain injury [39, 40] and that one-fifth of MCS patients may regain functional independence, with almost 18% capable of working [39, 41]. The recovery in these patients, when present, could be slow [17, 18]. In 6 months of observation, we found a change in the ratio between UWS/VS and MCS in our patients’ group. Initially, the UWS/VS patients were 58%, and after 6 months, 45%. However, in patients hospitalized for a long time, observing changes in behavioral responses may be less accurate because the assessment with the behavioral scales is less frequent.

Moreover, the less intensive rehabilitative intervention might make it challenging to detect eventual behavioral changes. Nevertheless, with an assessment per month, we found that the CRS-R total score and the WHIM TNB were positively correlated with the time of observation in the UWS/VS patients independently from the etiology. Furthermore, the same correlations were for the WHIM TNB visual and auditive clusters.

Our findings suggest that visual and auditive items of used scales could be predictors in the change of the consciousness levels, confirming the study of Lee [42] and colleagues, which suggest higher auditory, communication, arousal, and total CRS-R scores as important predictors of patients who emerged from PDOC.

In our sample, 5 UWS/VS change the level of consciousness in MCS between 184 and 394 days from the acute event (1 hemorrhagic, 2 traumatic brain injury, and 2 other etiology) and one (brain anoxia) after 1705 days. Three of these patients showed a marked increase in the CRS-R total score in the second assessment. This could be due to the accidental low arousal on the first assessment or other clinical onset, as well as to a spontaneous improvement of consciousness state.

It is interesting to note that in some patients (i.e., n.ro 10, 13, 17, 23, 30, and 39), the WHIM is constant or change from the first to the second assessment, whereas the CRS-R show more fluctuations. This is due to the intrinsic characteristic of the scoring in these scales. Since to assign an item with the WHIM is sufficient to observe a behavioral response, it could be already present in the WHIM but does not meet the criteria to be assigned in the CRS-R. This could produce a successive change in the CRS-R total score (because the criteria to assign the item are satisfied) but not in the WHIM (because the item was already present).

Furthermore, ten patients (25%) were hospitalized in the unit for a period ranging from 3 to 9 years. These results highlight the importance of assessing PDOC patients after a long time and that a continuous, also if not intensive, program of stimulation might induce a change in the level of consciousness.

To explain the progressive return of behavioral responsiveness across different levels of DOC, it was postulated that restoration of function within the anterior forebrain mesocircuit substantially correlates with activation of the frontoparietal network. This model, known as the “mesocircuit” model, points attention to the role of central thalamic neurons and their frontostriatal connection [43]. The recovery of consciousness depends on increasing metabolic activity and functional connectivity between the forebrain mesocircuit and frontoparietal network [44, 45], as well as on the functional recovery of the ascending reticular activating system [46]. A recent work [47] supports this model, evidencing how in PDOC patients is disrupted the function of the inhibitory role of the anterior forebrain mesocircuit on the Default Mode Network, which is involved, with the medial prefrontal cortex, posterior cingulate, and precuneus, in mediating the internal awareness [48]. In a recent study with high-density electroencephalography (hdEEG), Bareham and colleagues [16] evidenced that the decrease in theta power and increases in alpha connectivity are predictors of changes in CRS-R scores over time. These finds suggest that the improvements in functional brain networks could precede changes in the level of consciousness in PDOC patients. However, the late recovery of consciousness in PDOC patients was observed to depend on etiology, age, and time since the brain injury [17], which could influence the restoration of cerebral network activity.

In any case, the first 2 years following an injury were reported as critical because, in this range of time, the patients are most vulnerable to life-threatening complications [17, 18]. In our study, two of the assessed patients died 4 months after the study’s start. Both were UWS/VS affected by brain hemorrhage, but one died almost 1 year after the acute event and the second after 3 years. Five other patients (3 MCS and 2 UWS/VS) were transferred at home after 4 months after the study’s start. Of these, two UWS/VS and one MCS (all affected by brain anoxia) died within 1 year of discharge.

Predicting the longer-term prognosis of PDOC patients following brain damage is challenging for clinicians also because few studies have rigorously monitored patients’ recoveries. The difficulty of conducting longitudinal studies involving systematic follow-up of PDOC patients derives from transferring them to nursing homes, specialist neurological centers, or their families with consequent incomplete records of the clinical course and outcomes.

The length of time in observing patients with DOC is relevant to highlight changes in their consciousness level. Giacino describes a “nihilistic attitude” toward PDOC patients and their exclusion from rehabilitation in some health cultures and that the standard 6-week programs available in the USA are frequently incompatible with the course of recovery in this group [49]. On the contrary, the UK provides reasonably good care for PDOC patients. Indeed, before achieving a clinical diagnosis, the patients can have an average of 4 months of intense therapy in the acute environment, followed by 2–7 months of comprehensive examination under optimal settings [50].

In our institute, TBI patients undergo an intensive rehabilitation program until 1 year after the acute event, and patients with other etiology until 6 months. The patients who have not evolved from UWS/VS or MCS and are unsuitable for discharge or home care are transferred to a dedicated unit. Here, complete nursing and medical care, appropriate nutrition and hydration, wheelchair adaption, and passive motor therapy are all provided, and ad hoc procedures are used to track any potential progress toward a (partial) return of awareness. With the goal of reintroducing the patient to their home environment, the family is trained to care for them at home for brief periods of time when it is practical. It is also possible to extend the healthcare and neurorehabilitation at the patient’s home under remote control, thanks to a collaboration between our institute, the local government, and the healthcare organization [51].

The long stay in UWS/VS and MCS makes difficult the assessment of subtle changes in the consciousness level, and in this frame, the WHIM seems to be a useful assessment tool. The CRS-R was developed to diagnose patients with DOC with scoring based on standardized stimuli necessary to observe more times to assign the observed behavior. Instead of arranging stimuli and responses in a modality-based grouping of behaviors, the WHIM comprises 62 items of increasing complexity that occurred spontaneously or following stimulation during observation [52]. The modality of WHIM in observing patients with PDOC could help detect subtle changes in everyday life, helping identify short-term goals [27, 52, 53]. The subtle changes could be characterized by differences in spontaneous behaviors, such as increasing time in eye-opening, or different behavioral responses to environmental stimuli presented accidentally. These slight behavioral changes may not have a statistically significant meaning but may be significant in changing the approach with the patients.

It was observed that the serial WHIM evaluations that produced a trajectory of change also predicted 68% of the variation in PDOC status on discharge from inpatient rehabilitation [50], and that may be more sensitive to some signs of higher levels of consciousness than the CRS-R [54]. However, the WHIM is less clinically applicable than CRS-R because its outcome and diagnosis are not directly linked (i.e., the assessment is done without giving any diagnosis since that does not incorporate criteria to make a diagnosis of DOC) [55].

Dhamapurkar and colleagues [56] reported that regular WHIM assessments might identify early signs of infections in PDOC patients. They correlated this scale scores with the pre-infection period and with post-infection and observed that the decrease in WHIM scores was related to the infection insurgence. Turner-Stokes and colleagues highlight the diagnostic utility of the scale and that the trajectory of change is an outcome predictor, suggesting a new order for the WHIM items and proposing future studies [50].

Our findings confirm the Turner-Stokes results, showing the importance of the combined CRS-R and WHIM assessment in PDOC patients to have at the same time a diagnostic assessment and the possibility of tracking subtle changes. In fact, while the CRS-R was developed to diagnose UWS/VS, MCS, or emersion from MCS, the WHIM was developed to identify sequences of recovery processes. The different criteria in the scoring attribution and different modality in assessing the patients make the WHIM potentially more sensible in detecting subtle behavioral changes. Moreover, WHIM is generally simple to use in neurorehabilitation settings, requires less staff training, and although WHIM takes relatively more time to administer, it allows a regular or routine serial examination. These aspects could make WHIM useful to help therapists and relatives to identify which stimuli, in different environments (e.g., in the context of domiciliary care), work better for the patient [51].

The treatment approach, modality for observing and tracking behavioral changes in patients with PDOC, could play a crucial role in accurately assessing their level of consciousness. It should be noted that changes in their behavioral response may reflect a favorable clinical trend rather than simply a spontaneous recovery of consciousness or a variation in arousal.

Our sample allowed us to observe a slow change in the consciousness level in PDOC patients thanks to the number of patients, different etiology, and different time from the acute event (i.e., from 6 months to 9 years). However, the single-center study, one monthly assessment, and only 6 months of observation represent a limitation. A long-term follow-up and more monthly assessments could help better observe changes in consciousness levels in these patients and predict possible outcomes.

In conclusion, this study has demonstrated that the monthly assessment of PDOC by means of the CRS-R and WHIM was able to detect also subtle changes in consciousness level. Therefore, these tools should be more frequently administered in long-term care for a better management and a more tailored rehabilitation of these very frail and vulnerable individuals.
